# Genomically integrated orthogonal translation system in *Escherichia coli* enables production of functional modified [NiFe]-hydrogenases

**DOI:** 10.1186/s12934-026-03057-z

**Published:** 2026-07-06

**Authors:** Qin Fan, Stevanie Stevanie, Stefan Frielingsdorf, Peter Neubauer, Oliver Lenz, Matthias Gimpel

**Affiliations:** 1 Chair of Bioprocess Engineering, Institute of Biotechnology, Technische Universität Berlin, Ackerstrasse 76, 13355 Berlin, Germany; 2 Institute of Chemistry, Biophysical Chemistry, Technische Universität Berlin, Straße des 17. Juni 135, 10623 Berlin, Germany

**Keywords:** CRISPR/Cas9, [NiFe]-hydrogenase, *Cupriavidus necator*, Orthogonal translation system (OTS), Genomic integration, Genetic code expansion

## Abstract

The functional diversification of O_2_-tolerant [NiFe]-hydrogenases using orthogonal translation systems (OTSs) offers a promising strategy for developing advanced biocatalysts and biohybrid energy platforms. However, plasmid-based OTSs frequently impose metabolic burdens and suffer from plasmid instability during fermentation, particularly when co-produced with complex metalloenzymes. To overcome these bioprocess limitations, we employed CRISPR/Cas9-mediated genome editing to integrate a psychrophilic pyrrolysyl-tRNA synthetase/tRNA pair into the *Escherichia coli* BL21 genome. The resulting strain provided a plasmid-free orthogonal translation background that supported amber suppression-mediated expression of the regulatory [NiFe]-hydrogenase (RH) of *Cupriavidus necator*. Using this genomically integrated OTS, we achieved the production of a full-length, catalytically active RH variant. Our results demonstrate that chromosomal OTS is compatible with the efficient production and maturation of complex metalloenzymes. The present work lays the groundwork for the bio-orthogonal engineering of hydrogenases and related hybrid biocatalysts.

**Supplementary Information:**

The online version contains supplementary material available at 10.1186/s12934-026-03057-z.

## Introduction

O_2_-tolerant [NiFe]-hydrogenases are metalloenzymes that catalyze the reversible conversion of molecular hydrogen (H_2_) into protons and electrons in the presence of O_2_, which makes them attractive for a range of biotechnological applications, including H_2_ production, bioanodes in biofuel cells and systems for H_2_-driven cofactor regeneration [1–6]. However, the limited yield of active hydrogenase from their native as well as heterologous microbial hosts poses a significant challenge for both technical applications and fundamental research [7–9]. Recently, a bioprocess for the heterologous production of hydrogenases has been developed in a robust *Escherichia coli* strain, demonstrated using the O_2_-tolerant regulatory [NiFe]-hydrogenase (RH) of *Cupriavidus necator* (formerly *Ralstonia eutropha*) as a model enzyme (Figure S1). This approach resulted in a 130-fold increase in yield of catalytically active hydrogenase compared to native production [10–12], demonstrating the feasibility of heterologous production of active hydrogenase in *E. coli* with high yields, short process times, and good scalability, thereby greatly reducing production costs [12, 13]. 

The electron-donating capacity of hydrogenases based on clean fuel H_2_ has sparked interest in using click chemistry to develop H_2_-driven coupled chemo-bio catalysts. However, to effectively harness this catalytic power in hybrid systems, precise interfacing between the enzyme and non-natural redox partners or electrode surfaces is required. Site-specific incorporation of clickable non-natural amino acids (ncAAs) provides a unique bio-orthogonal “anchor” for such interfacing, allowing for controlled orientation and efficient electron transfer. To enable such site-specific chemical modifications, it is essential to establish a biological system capable of incorporating ncAAs with the desired properties for the subsequent coupling into hydrogenases in vivo. A common approach for site-specific ncAA incorporation is amber stop codon suppression, which reprograms the UAG stop codon to encode a ncAA [14–17]. This process relies on the activity of an orthogonal translation system (OTS) consisting of an ncAA-specific aminoacyl-tRNA synthetase (aaRS) that specifically attaches the ncAA to the cognate tRNA, and a suppressor tRNA that decodes the amber codon [18–20]. These OTS components are typically encoded on plasmid vectors [21, 22]. The recently developed “cold”-adapted pyrrolysyl-tRNA synthetase (PylRS) from the psychrophilic archaeon *Methanococcoides burtonii* (*Mbur*PylRS) shows outstanding ncAA incorporation efficiency and the required substrate promiscuity. Together with the engineered tRNA from *M. alaskense*, the modified *Mbur*PylRS variant proved to be extremely efficient in incorporating S-allyl-cysteine (Sac), the smallest clickable ncAA for click chemistry, at up to 5 sites into a target protein with good yield, even at very low Sac concentrations [23–26]. 

However, plasmid-based expression systems often encounter significant challenges, such as high metabolic burden imposed by antibiotic effects and plasmid maintenance, and plasmid instability during large-scale cultivation. In the specific case of [NiFe]-hydrogenase, genetic stability is paramount, as the maturation process is exceptionally complex and, in addition to the *hoxN* gene for efficient nickel transport, the process also requires the coordinated co-expression of seven *hyp* genes for cofactor assembly [11]. Ensuring consistent and stable OTS expression is therefore crucial for efficient ncAA incorporation in protein synthesis and for maintaining the resilience of synthetic cells across different environments.

Hence, there is a significant need for the stable and safe integration of OTS components into the host genome [16, 27]. This necessitates an efficient chromosomal editing method for precise localization and long-term genetic stability. The most popular CRISPR/Cas-mediated genome editing offers a promising approach for safe and accurate integration of orthogonal pairs into the bacterial chromosome. The system can generate a Cas9-mediated double-strand break (DSB) at almost any desired target DNA locus [28–30]. Since the DSB is lethal, only cells for which the target sequences have been edited, i.e. through homology-directed repair (HDR) with exogenous homologous donor DNA, can survive [31–33]. These systems can be coupled with the λ-red system to accomplish efficient editing of the *E. coli* genome [34–37]. Even though, the integration efficiency and accuracy of these methods are mainly limited by off-target effects and other integration byproducts [33, 38–41]. Recently developed simple CRISPR/Cas9-assisted gRNA-free one-step genome editing technique (CAGO) utilizes a universal gRNA guided by a universally designed N20 sequence for increased targeted editing efficiency [42]. The required editing elements are encoded on the helper plasmid pCAGO. This technique simplifies the editing process, requiring only the transformation of the editing strain and the construction of an editing cassette carrying an antibiotic resistance gene for selection. In a first recombination event catalyzed by the λ-red system, the editing cassette containing a selection marker, the universal N20 PAM and the gene of interest, is integrated in the genome. Subsequently, the resistance gene can be removed following a Cas9 induced double strand break at the introduced N20 PAM site and a 2nd λ-red mediated recombination [42]. 

In this study, we harnessed the CAGO technique to integrate the psychrophilic PylRS/tRNA pair directly into the chromosome of *E. coli* BL21 Gold. This strain was selected because it has been extensively validated in our previous bioprocess developments as a robust platform for high-yield heterologous production of catalytically active [NiFe]-hydrogenases, enabled by the co-expression of the maturation machinery required for the correct hydrogenase assembly [10–12]. By embedding the OTS within the host genome, we achieved successful production of full-length bio-orthogonal modified RH variants. This work addresses the bottlenecks observed in plasmid-based OTS approaches, offering a new perspective for the production of bio-orthogonally modified hydrogenases.

## Materials and methods

### Strains, media and growth conditions

*E. coli* TG1 [43] was used for cloning, while BL21 Gold (Stratagene, Germany) was used for the genome editing experiments and protein expression. TY medium (16 g L^− 1^ tryptone, 10 g L^− 1^ yeast extract, 5 g L^− 1^ NaCl) or EnPressoB medium (EnPresso GmbH, Germany) was used for cultivation. For selection purposes, 125 µg mL^− 1^ ampicillin, 34 µg mL^− 1^ chloramphenicol and 50 µg mL^− 1^ kanamycin (final concentrations) were added. The genes encoding the RH subunits HoxB and HoxC were expressed from plasmid pQF8 (native HoxB, HoxC) [10], pQF26 (HoxB(N223*), HoxC) and pQF27 (HoxB(T250*), HoxC) without OTS genes, or pQF28 (HoxB(N223*), HoxC) and pQF29 (HoxB(T250*), HoxC) with OTS genes. In all cases, genes encoding the RH maturation enzymes HypA1B1F1CDEX and the *C. necator* nickel permease HoxN were co-expressed from plasmid pQF18 [11]. All strains and plasmids are listed in Supplementary Tables S1 and S2, respectively.

### Plasmid construction

The high copy number plasmid pQF8 [10] was used for overproduction of the RH subunits HoxB_Strep_ and HoxC. Incorporation of the amber stop codon at positions in the *hoxB* gene corresponding to N223 and T250 in the HoxB protein was achieved by oligonucleotide-based site-directed mutagenesis with two sequential PCR reactions. The *hoxB*(N223*) allele was constructed using the primer pairs MG358/MG47a and MG38/MG359 (all oligonucleotides are listed in Supplementary Table S3) and plasmid pQF8 as PCR template. The two resulting amplificates (715 bp and 711 bp) were used as template for a second PCR using primers MG47a/MG38. The resulting product was digested with XbaI and BstBI, and the resulting 922 bp fragment was ligated into plasmid pQF8, which had been cut with the same enzymes, yielding plasmid pQF26, encoding the RH variant with HoxB(N223*). Plasmid pQF27, encoding the RH variant with HoxB(T250*) was generated in the same manner, using the primer pairs MG360/MG47a and MG38/MG361 for the first PCR and the primer pair MG47a/MG38 for the second PCR. The genes of the orthogonal translation system (OTS) for Sac incorporation, consisting of *M. burtoniii* pyrolysyl tRNA synthetase (*Mbur*PylRS) and *M. alaskense* tRNA_CUA_ under control of the constitutive promoters *lpp* and *proK*, respectively, were obtained from plasmid pNK552 [44]. The OTS genes were PCR amplified using primers MG364/MG365 and plasmid pNK552 as template. The resulting 1.8-kb fragment was digested with SacI, and the resulting 1793-bp fragment was then ligated into SacI-cut pQF26 and pQF27, yielding plasmids pQF28 and pQF29, respectively. The correctness of all inserts was confirmed by DNA sequencing.

### Generation of the linear editing donor cassette

The linear editing donor cassette was generated using Golden Gate assembly (Figure S2, step 1). First, four fragments, each containing a BsaI restriction site that produces a specific 4-bp overhang upon cleavage, were PCR amplified using Q5 DNA polymerase (New England Biolabs). Fragments 1 and 4 representing the left (L-arm) and right homology arms (R-arm) required for subsequent recombination with either SS3 or *lacZ*, were amplified using *E. coli* BL21 chromosomal DNA and the primer pairs shown in Figure S2, step 1 (primers are listed in Table S3). The chloramphenicol resistance gene was amplified from plasmid pQF3 [10] using the primer pair QF3/QF21. The universal N20PAM sequence (TACTTCGGTTCGATGGACTA) required for CRISPR/Cas9 editing was embedded in the reverse primer QF21. The PylRS/tRNA genes were amplified from plasmid pNK552 using the primer pairs QF5/QF22 and QF15/6 for SS3 and *lacZ*, respectively. A 40-bp L-short sequence, long enough to ensure sufficient editing efficiency [42], was embedded in the forward primers QF5 and QF15. For Golden Gate assembly, approximately 200 ng of each fragment was added to the 20 µL (total volume) reaction mixture, which also contained T4 ligase buffer, 1.5 µL T4 DNA ligase (600 U) (New England Biolabs), and 1 µL BsaI (20 U) (New England Biolabs). The assembly reaction was performed in a thermocycler with 60 cycles (5 min at 37 °C, 5 min at 16 °C) followed by final incubation at 37 °C for 5 min and inactivation of the enzymes at 60 °C for 5 min. The assembled DNA editing cassette was purified via gel extraction using the NucleoSpin^®^ Gel and PCR Clean-up Kit (Macherey-Nagel, Germany), and then amplified by PCR with the primer pairs QF1/8 and QF13/17 for SS3 and *lacZ*, respectively (Figure S2, step 2). The amplified PCR products were separated electrophoretically in agarose gels, excised, and subsequently purified by gel extraction.

### CAGO genome editing procedure

10 mL TY medium with ampicillin were inoculated in a 1:100 ratio with *E. coli* BL21 Gold harboring plasmid pCAGO from an overnight TY preculture. At an OD_600_ of approx. 0.2, production of the λ-red proteins (γ, β, and exo genes from bacteriophage λ) was induced by adding 1 mM IPTG. The cultivation was continued at 37 °C and 250 rpm until an OD_600_ of 0.8–1.0 was reached. Cells were harvested by centrifugation, resuspended in 600 µl ice-cold CaCl_2_ solution (0.1 mM CaCl_2_, 50 mM Tris-HCl, pH7.5) and incubated on ice for at least 2 h to make them chemically competent. For transformation, 100 µl of competent cells were mixed with approx. 600 ng of the editing cassette. The cells were incubated on ice for 30 min, heat-shocked for 2 min at 37 °C and cooled on ice for 3 min. After adding 1 mL TY medium and incubating with gentle shaking at 30 °C for 2 h, the transformed cells were spread onto TY agar plates containing ampicillin, chloramphenicol, and 1% glucose to suppress leaky expression of Cas9 and λ-red (Figure S2, step 3). Single colonies were picked and recombination of the editing cassette at the target sites was verified by colony PCR and subsequent sequencing (LGC Genomics, Germany). Positive clones were incubated in 10 mL TY medium supplemented with ampicillin, and the expression of the λ-red system and Cas9 was induced with 1 mM IPTG and 10 mM L-arabinose, respectively (Figure S2, step 4). After overnight cultivation at 30 °C, appropriate dilutions (10^− 7^–10^− 8^) were spread onto TY agar plates with ampicillin, which were incubated overnight at 37 °C (Figure S2, step 5). To identify correctly edited clones, single colonies were picked and the removal of Cm^R^ was verified by antibiotic sensitivity, colony PCR, and subsequent sequencing. The editing efficiency was calculated as the ratio of the number of colonies with correct colony PCR product to the number of colonies tested on the antibiotic selection plates.

### Plasmid curing

Clones containing the correct genome editing cassette were cured of the temperature sensitive pCAGO plasmid. Colonies were picked and streaked onto antibiotic-free TY plates, which were incubated at 42 °C overnight. Each colony resulting from this selection procedure was transferred to two distinct TY plates with and without 125 µg mL^− 1^ ampicillin. Both plates were incubated overnight at 37 °C and the colonies sensitive to ampicillin were confirmed by antibiotic sensitivity to be pCAGO-free. The final strain was designated *E. coli* BPylRS1 (*E. coli* BL21 containing the chromosomal PylRS/tRNA OTS).

### Hydrogenase production, purification and activity assay

*E. coli* strains BQF8RH8 [10], BPyl26RHN8 (*E. coli* ByplRS1 (pQF26, pQF18)), BPyl27RHT8 (*E. coli* ByplRS1 (pQF27, pQF18) and BQF28RHN8 (*E. coli* BL21 Gold (pQF28, pQF18)) and BQF29RH8 (*E. coli* BL21 Gold (pQF29, pQF18)) were used to evaluate RH production and ncAA incorporation into HoxB. Cultivations on a shake-flask scale were carried out in 250 mL Ultra Yield Flasks (UYFs) filled with 50 mL EnPresso B medium, as described previously [10, 11]. After overnight EnPresso cultivation, booster and 4.5 U L^− 1^ enzyme were added, and RH production induced with 50 µM IPTG and 2 mM Sac (TCI America) for ncAA incorporation. For RH metal center assembly, 0.1 mM NiSO_4_, 0.1 mM FeCl_3_ were also added. Cultivation was continued at 18 °C for 48 h. Cells were harvested by centrifugation at 8000x* g* and 4 °C for 10 min, and the cell pellets were immediately frozen at –80 °C until further use.

RH purification via Strep-Tactin affinity chromatography was performed as previously described [10, 11, 45]. Aliquots of the elution fractions were subjected to SDS-PAGE analysis. Bovine serum albumin (BSA) served as standard for the determination of protein concentrations, and Roti-Mark TRICOLOR (Carl Roth) was used as a protein marker. The gels were stained with colloidal Coomassie blue G250 solution and the protein bands were quantified using the ImageJ software. All elution fractions were pooled and concentrated using Amicon Ultra Ultracel concentrator with a 10 kDa cut-off (Merk Millipore, Germany) according to the manufacturer’s instructions. The spectrophotometric determination of the H_2_-oxidation activity was performed as previously described [11]. Measurements were performed with two biological replicates.

## Results and discussion

### Selection of site-specific TAG mutation positions and construction of expression vectors for PylRS/tRNA pair in *E. coli*

**Fig. 1:**
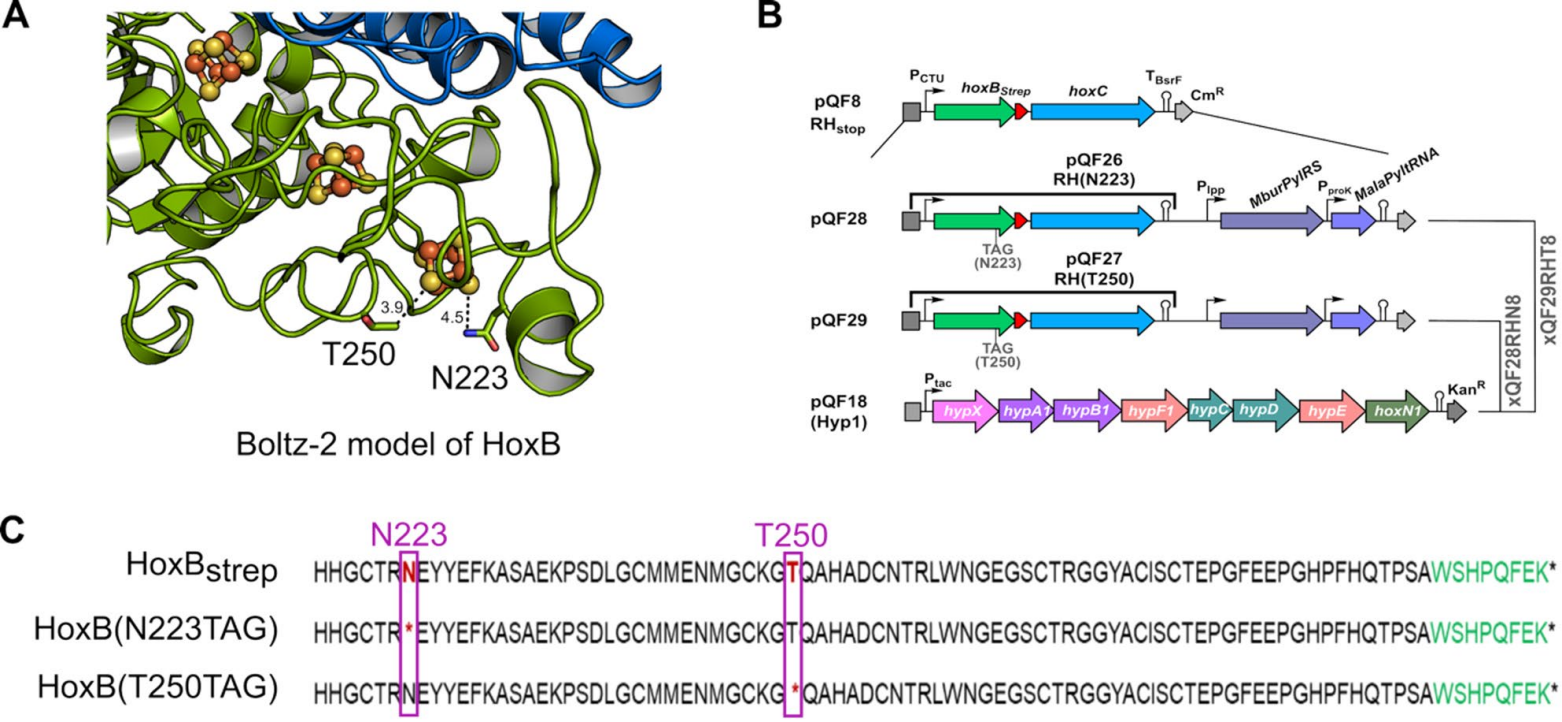
Bio-orthogonal labeling of the HoxB subunit of the *C. necator* RH via in-frame amber stop codon suppression. **A** Boltz-2-predicted structural model of HoxB (green), which carries three [4Fe-4 S] clusters. The selected ncAA incorporation sites T250 and N223, as well as their distances from the distal [4Fe-4 S] cluster, are highlighted. **B** Plasmids and strains used for production of RH variants via amber suppression. Key genetic elements such as psychrophilic PylRS/tRNA system components and incorporation sites are annotated. **C** Amino acid sequences of the C-termini of the native and modified HoxB variants, with the positions for amber codon introduction and ncAA incorporation highlighted. The C-terminal Strep-tag II sequence is shown in green letters

The first step in introducing ncAAs through engineered orthogonal pairs of aminoacyl-tRNA synthetases and tRNAs is to identify suitable positions in the target protein. The RH of *C. necator* consists of the large subunit HoxC containing the catalytic [NiFe] center and the small subunit HoxB carrying three [4Fe-4 S] clusters in a row, which shuttle the electrons released by the catalytic center to the primary electron acceptor (Figure S1). In order to harness the electrons released during H_2_ oxidation, the ncAA, to which a potential redox catalyst can be coupled, should be installed near the electron release site of HoxB, which is the distal [4Fe-4 S] cluster. Furthermore, the incorporation of the ncAA must not impair either the structure or the catalytic activity of the RH. Given these guidelines, two positions within the C-terminal region of HoxB, Asn223 and Thr250, were selected as sites for ncAA installation based on an in silico HoxB structure predicted by Boltz-2 (Fig. 1A) [46]. The spatial distances of N223 and T250 from the distal [4Fe-4 S] cluster are approx. 4.5 Å and 3.9 Å, respectively. These values are well within the generally accepted ~ 14 Å limit for efficient biological electron transfer [47] and significantly shorter than the average spacing between the native Fe-S clusters or between the [NiFe] active site and the proximal [4Fe-4 S] cluster (< 10 Å) [48, 49]. Therefore, incorporation of the ncAA and subsequent attachment of a redox catalyst through click chemistry would place the catalyst within a highly permissive range of electron transfer distance with the distal [4Fe-4 S] cluster. To validate that the selected sites are accessible for post-translational modification, we calculated the Solvent Accessible Surface Area (SASA) and Relative Solvent Accessibility (RSA) based on the Boltz-2 model using PyMol (Figure S3). With values of 0.35 and 0.23, the selected amino acids N223 and T250, respectively, have RSA values above the threshold of 0.2 for solvent exposure, indicating that they are not buried within the protein fold. This indicates that the ncAA incorporated at these positions should be spatially accessible for subsequent click chemistry.

Consequently, the *hoxB* codons for N223 and T250 in plasmid pQF8 [10] were individually replaced with amber stop codons resulting in plasmids pQF26 (N223*) and pQF27 (T250*) (Fig. 1B and C, S4). Both HoxB variants carry a C-terminal Strep-tag (Fig. 1C), which allows for simple purification of ncAA-containing RH variants via affinity chromatography after amber codon suppression. Subsequently, the genes of an OTS, comprising the pyrrolysyl-tRNA synthetase (PylRS) from the psychrophilic *Methanococcoides burtoniii* and the engineered tRNA from *M. alaskense*, were inserted into plasmids pQF26 and pQF27, resulting in plasmids pQF28 and pQF29, respectively. Instead of the widely used OTSs derived from mesophilic or thermophilic organisms, we selected an OTS from a psychrophile, as the RH production process takes place at 18 °C to enable correct and more efficient RH maturation, i.e. metal cofactor insertion [11]. 

### Production of ncAA-labeled RH variants in plasmid-based OTS system

The size of the ncAA to be incorporated is of crucial importance. An excessively long side chain not only carries the risk of unexpected structural and functional changes due to its greater flexibility [50, 51], but also increases the distance between the distal [4Fe-4 S] cluster and the catalyst to be clicked, which could impair electron transfer. To mitigate these constraints, we selected Sac, for integration into RH.

To evaluate Sac compatibility of the OTS, the plasmids pQF28/29 (harboring the RH structural and OTS genes) and pQF18 (maturation genes) were transformed into *E. coli* BL21 Gold, and the resulting strains BQF28RHN8 and BQF29RHT8 were cultivated in EnPresso B medium supplemented with different Sac concentrations as substrate for ncAA incorporation. Unfortunately, even at a high Sac concentration of 10 mM, no assembled Strep-tag II-tagged RH variants could be purified from the soluble extracts of *E. coli* BQF28RHN8 and BQF29RHT8 cells using Strep-Tactin affinity chromatography (Figure S5A). Immunological analysis of the flow-through using anti-HoxB antibodies showed only truncated HoxB species, indicating premature translation termination at the amber codon rather than Sac incorporation (Figure S5B). High-copy number plasmids are known to burden the host cell metabolism, which can lead to cellular stress response and impaired tRNA charging, ultimately compromising ncAA incorporation efficiency [52–54]. This metabolic burden is particularly pronounced in the context of [NiFe]-hydrogenase production, which already requires the metabolic capacity of the host BL21 to support a multi-plasmid expression and maturation system. A genomically integrated system might provide a more stable genetic background that mitigates metabolic fluctuations associated with plasmid-based systems. Its lower expression levels and higher genetic stability might ensure a controlled supply of the Sac-charged tRNA and proper interaction with endogenous metabolic processes, thereby facilitating more efficient amber suppression at the UAG codon in the robust BL21 background [55–59]. 

### Selection of integration sites and generation of linear DNA editing cassettes

To incorporate the OTS into the chromosome of *E. coli* BL21 Gold, a suitable location for integration must be selected. Suitable sites for donor DNA editing cassettes have been classified as safety sites (SS) if they are located in an intergenic region, or as integration sites (IS) if they are designed to disrupt a genetic element (promoter, coding sequence or operon) [60]. We selected the *lacZ* gene and the intergenic SS3 [60] as OTS integration sites, considering blue-white screening for easy clone selection based on edited *lacZ* and the high integration efficiency (100%) at SS3. For genome editing, the recently developed RNA-guided CRISPR/Cas9-based genome editing approach (CAGO) [42] was used. This system was specifically chosen for its capacity to achieve scarless genomic editing [42] in order to retain the antibiotic selection markers for the plasmids required for functional hydrogenase production. Maintaining these markers is essential, as hydrogenase expression relies on a multi-plasmid system. To avoid potential off-target effects, Cas-OFFinder [61] was used to confirm that the selected N20 sequence (5’-TAGTCCATCGAACCGAAGTA, N20PAM, Figure S2) has no complementary sequence in the genome of *E. coli* BL21. Eight sequences with six mismatches and one sequence with five mismatches (Table S4) showed the highest similarities, which are therefore unlikely to represent off-target positions. Thus, the selected N20PAM should be suitable for editing *E. coli* BL21 Gold. The amplification of the required fragments of the linear editing cassette, i.e. left homology arm, right homology arm, a 40-bp fragment (L-short, homologous to the left homology arm) coupled to the *PylRS*/tRNA pair and the antibiotic resistance cassette with the N20PAM recognition site (Figure S2) was confirmed by agarose gel electrophoresis (Figure S6A), and the fragments were then successfully linked together by Golden Gate assembly (Figure S6B).

### Transformation of plasmid pCAGO for CRISPR/Cas9

High transformation efficiency is crucial for successful CAGO genome editing, as the linear DNA editing cassette must be efficiently taken up by the host cells to obtain sufficient numbers of correctly edited clones. This requirement is particularly critical for *E. coli* B strains (e.g. BL21 derivatives), which are widely used as production hosts for high-level heterologous protein synthesis, but less frequently the focus of genome-editing method development than K-12 derivatives and often display lower or more variable transformation efficiencies under conditions optimized for K-12 strains [62, 63]. For *E. coli* B strains it is therefore important to optimize the conditions for the production of chemically competent cells in order to overcome strain-specific limitations in DNA uptake and to achieve transformation efficiencies compatible with the robust recovery of correctly edited clones in CRISPR-based technique. The exact relationship between growth stage and chemical competence may vary depending on the specific *E. coli* strains and growth conditions [64, 65]. Our systematic investigations for *E. coli* BL21 Gold cells revealed a maximum competency at an OD_600_ of 1.0–1.2 (Figure S7A, B). At OD_600_ values significantly exceeding 1.0, no further improvement of transformation yield was observed, which is likely due to a decrease in the number of cells capable of taking up the exogenous genetic material. On the other hand, transformation of cells prepared from the early exponential phase (OD_600_ of 0.5) resulted in almost no colonies. This contrasts markedly with the results obtained with *E. coli* K-12 strains, where the highest transformation efficiency is achieved with cultures harvested at OD_600_ of 0.3–0.5 [63, 64]. Nevertheless, even the highest transformation efficiency of about 6 × 10³ for *E. coli* BL21 Gold at an OD_600_ of 1.0 is still about 2–3 orders of magnitude lower than that of *E. coli* K-12 strains, confirming the poorer transformability of *E. coli* BL21 [66]. Nevertheless, an OD_600_ of approximately 1.0 appears to be optimal for the production of chemically competent *E. coli* BL21 Gold cells using the CaCl_2_ method.

Typically, a brief heat shock (30–120 s) at 42 °C is applied to facilitate DNA uptake by CaCl_2_-treated competent *E. coli* cells [67–70]. Consistent with the temperature-sensitive R101 *ori* [42], plasmid pCAGO could only be successfully transformed into *E. coli* BL21 Gold cells using a 2-minute heat shock at 37 °C (Figure S7C, D), even a brief 10-second heat shock at 42 °C resulted in no colonies at all (Figure S7D). In contrast, efficient transformation of the standard plasmid pUC19 was achieved by a heat shock of 10 s (or longer) at 42 °C (Figure S7D). Obviously, the R101 origin of pCAGO is extremely temperature-sensitive, which prevents plasmid replication at higher temperatures [71, 72] and even impairs plasmid uptake during transformation. Furthermore, pCAGO has a low copy number and large size, which also contributes the observed low transformation efficiency. Despite the low transformation efficiency with pCAGO, sufficient recombinant *E. coli* BL21 Gold colonies were obtained for subsequent strain engineering.

### Genome engineering

**Fig. 2:**
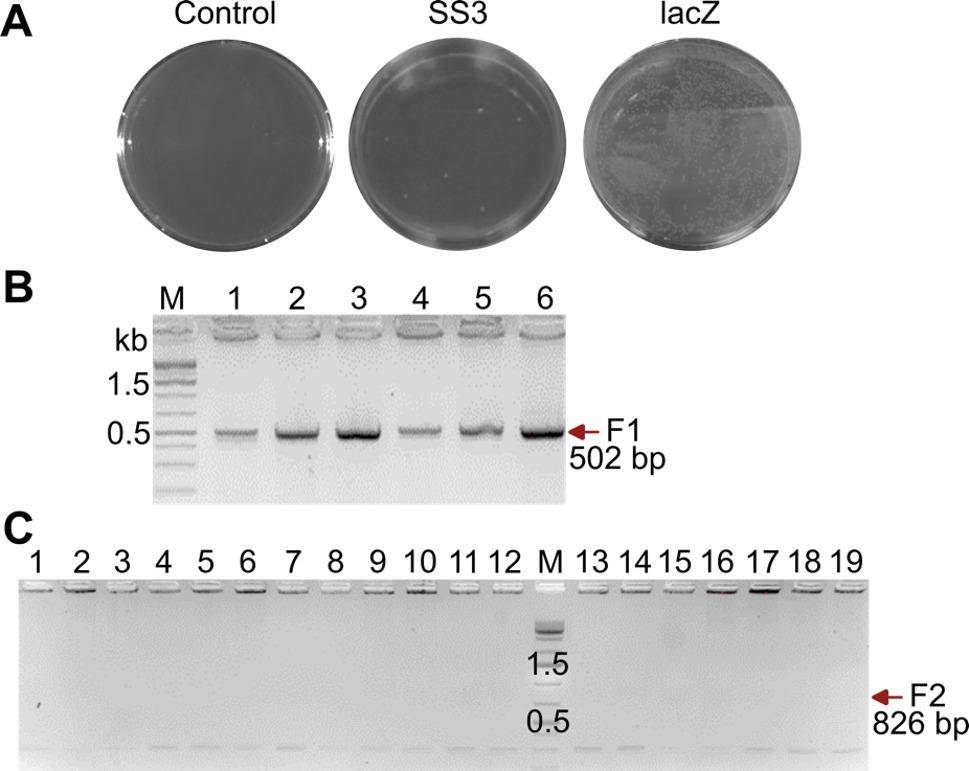
Integration of the linear DNA editing cassette into different sites of the *E. coli* BL21 Gold chromosome. *E. coli* BL21 Gold containing helper plasmid pCAGO was transformed with 600 ng of linear donor DNA editing cassette. **A** Colonies grown on TY_Amp_ and TY_Cm_ agar plates after activation of pCAGO. The entire transformation mixture was plated and incubated overnight at 37 °C. A transformation without linear donor cassette served as control. **B** Agarose gel electrophoresis after colony PCR using primer pair MG366/MG368 to verify the integration of the linear DNA cassette into SS3. The expected size of the fragment of 502 bp is indicated by a red arrow. **C** Agarose gel electrophoresis after colony PCR using primer pair QF10/QF18 to verify the integration of the linear DNA cassette into *lacZ*. The expected size of the fragment of 826 bp is indicated by a red arrow

600 ng of the linear editing cassettes were transformed into *E. coli* BL21 Gold cells overexpressing λ-recombinase genes and plated on agar plates with chloramphenicol and ampicillin antibiotics (Fig. 2A). Colonies that develop Cm resistance during selection should have integrated the editing cassettes successfully into their chromosome. Surprisingly, after overnight incubation only six clones were obtained after integration into SS3, whereas countless colonies were obtained from the presumed integration into the *lacZ* gene (Fig. 2A). The fact that no colonies appeared in the control without a donor cassette for HDR (Fig. 2A) shows that the selection process works in principle. The correct integration of the editing cassettes was then tested by colony PCR with primers that bind inside and outside of the cassette as well as exclusively at the target editing sites. The PCR product of all six SS3 transformants showed a band corresponding to the expected size of 502 bp (Fig. 2B). In contrast, none of the randomly picked transformants with the anticipated integration into *lacZ* showed a fragment (Fig. 2C), suggesting that the editing cassette was not present at the expected location. The apparent off-target integration could be due to unexpected non-specific integration events that were not predictable based on the primers used for the corresponding editing cassette. Although CRISPR/Cas9 is widely regarded as a precise genome-editing tool, multiple studies have shown that unintended edits (including off-target integrations and complex on-target rearrangements) can occur at low but detectable frequencies [73, 74]. Such outcomes appear to be more frequent or more readily recovered when targeting essential genes or when large repair templates are used, where selective pressure against deleterious on-target alleles can favor recovery of clones with atypical editing outcomes at alternative loci. Our observations are consistent with previous reports that genome editing can yield rare, unpredictable integration events [38, 73, 75]. The exact cause of the failure at the *lacZ* editing site remains unclear and was not further investigated in this study. Thus, we continued working only with the transformants carrying insertions in the SS3 site.

### Removal of the chloramphenicol resistance gene and pCAGO

**Fig. 3:**
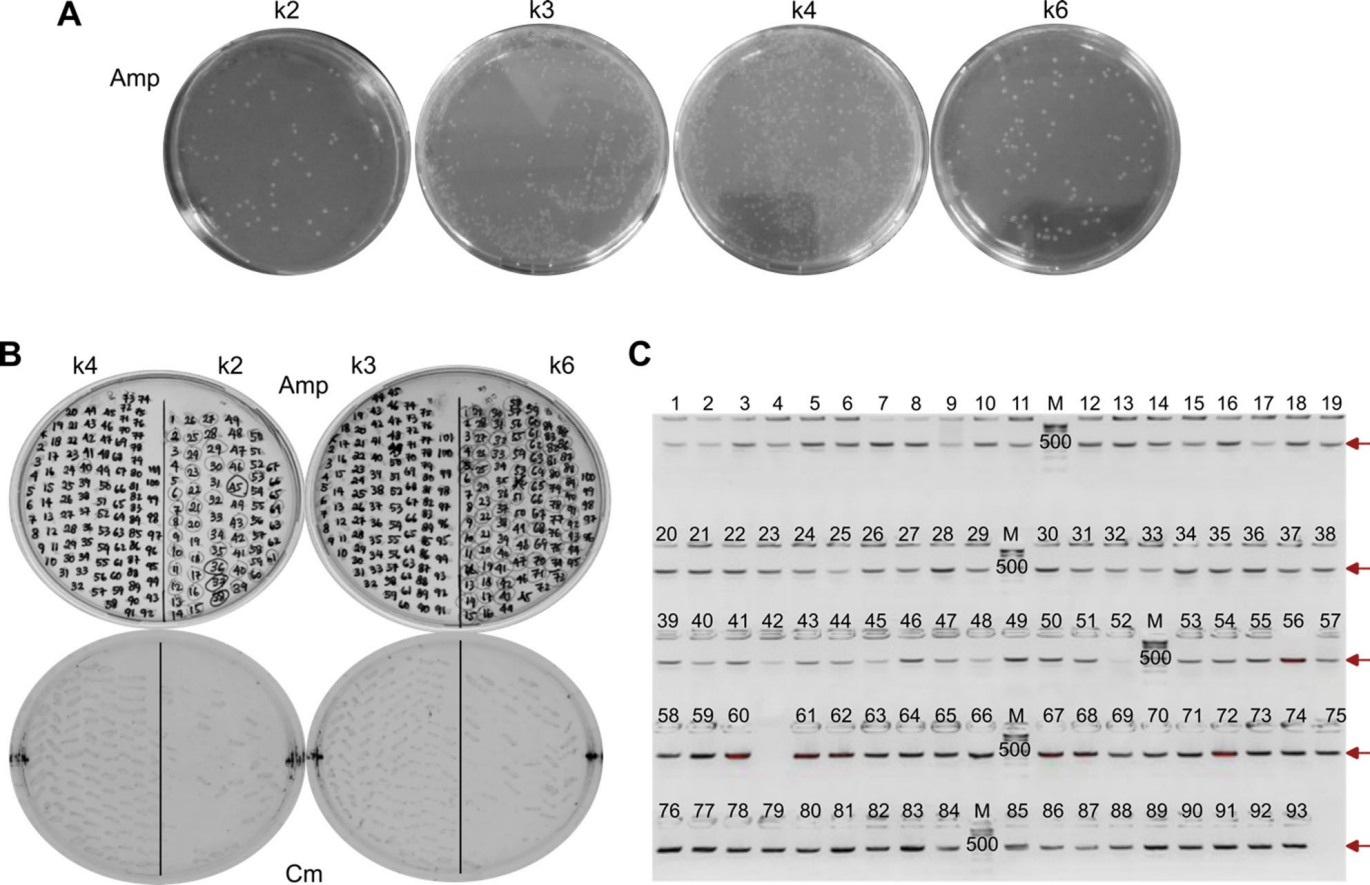
Removal of the Cm resistance gene from the genome. **A** The four clones k1, k3, k4, and k6, with integrated linear editing cassettes were cultivated in TY medium supplemented with 10 mM L-arabinose and 1 mM IPTG to induce CRISPR/Cas9 and the λ-red system, respectively. After overnight cultivation, the cells were diluted to 10^− 8^ with TY medium and spread onto TY_Amp_ agar. **B** Colonies were streaked on TY_Ap_ and TY_Cm_ agar to identify Cm-sensitive clones, indicating the successful removal of Cm^R^ gene from the chromosome. **C** Agarose gel electrophoresis of colony PCR of Cm-sensitive clones. Colony PCR was performed using primer pair QF12 and MG369. The expected size of the resulting product is 447 bp

The presence of the Cm^R^ gene in the genome after the initial genome engineering described above reduces the number of available selection markers and would interfere with the Cm^R^ gene of the plasmid encoding the structural genes of the *C. necator* RH. Thus, the genomic Cm^R^ gene was removed by introducing a CRISPR/Cas9 mediated dsDNA break followed by λ-red assisted HDR (Figure S2, step 4.1 and 4.2). The four clones k2, k3, k4, and k6, carrying the editing cassette at the SS3 site were cultivated in TY medium, and the λ-red system and CRISPR/Cas9 encoded by pCAGO were induced with 1 mM IPTG and 10 mM L-arabinose, respectively. After overnight cultivation, the diluted suspensions were spread on TY agar plates containing ampicillin (Fig. 3A). To verify successful recombination, single colonies were picked and streaked onto agar plates containing chloramphenicol to identify Cm-sensitive clones (Fig. 3B). Surprisingly, all selected clones derived from transformants k3 and k4 still grew in the presence of Cm, suggesting that either the CRISPR/Cas9 or the λ-red recombination system did not function properly (Fig. 3B). In contrast, 37 from 67 selected clones derived from transformant k2 and 71 from 100 clones of transformant k6 were Cm-sensitive, indicating (partially) successful genome editing. Evidence of successful removal of the Cm^R^ gene was provided by colony PCR, as all Cm-sensitive clones showed the expected 447-bp band upon agarose gel electrophoresis (Fig. 3C). Finally, the accurate insertion of the orthogonal PylRS/tRNA sequence at the SS3 site was verified by Sanger sequencing of two positive clones (Figure S8).

In summary, the genome editing efficiency we achieved (55–71%) is slightly lower than that reported for *E. coli* K-12 strains using the CAGO approach (75–100%) [42]. The lower editing efficiency might be due to the fact that, unlike *E. coli* K-12, *E. coli* BL21 lacks a functional Lon protease, which normally degrades the cell division inhibitor SulA induced by DNA damage [36, 76, 77] Once the DNA damage has been repaired, SulA is rapidly degraded by the Lon protease, allowing cell division to resume. In *E. coli* BL21 cells, SulA is more stable and cell division cannot resume as quickly after DNA repair [77, 78]. 

Finally, the helper plasmid pCAGO had to be removed from the engineered strain to ensure complete elimination of the CRISPR/Cas9 machinery, prevent unintended genome editing and obtain a completely marker-free strain. We randomly selected 18 clones that were streaked on TY agar plates with and without ampicillin followed by incubation at 42 °C overnight (Figure S9A). Despite the temperature-sensitive R101 *ori* of pCAGO, all colonies still grew in the presence of ampicillin, indicating that the plasmid loss was not yet confirmed at this stage. After re-streaking and a second overnight incubation at 37 °C, all 18 clones were sensitive to ampicillin, indicating the absence of pCAGO (Figure S9B). Thus, the CAGO approach enabled the successful integration of an OTS into the genome of *E. coli* BL21. The final and first *E. coli* BL21 Gold strain containing a chromosomally integrated orthogonal PylRS/tRNA system (without Cm^R^ and pCAGO), was named BPylRS1.

#### Functional evaluation of the genome-integrated OTS

**Fig. 4:**
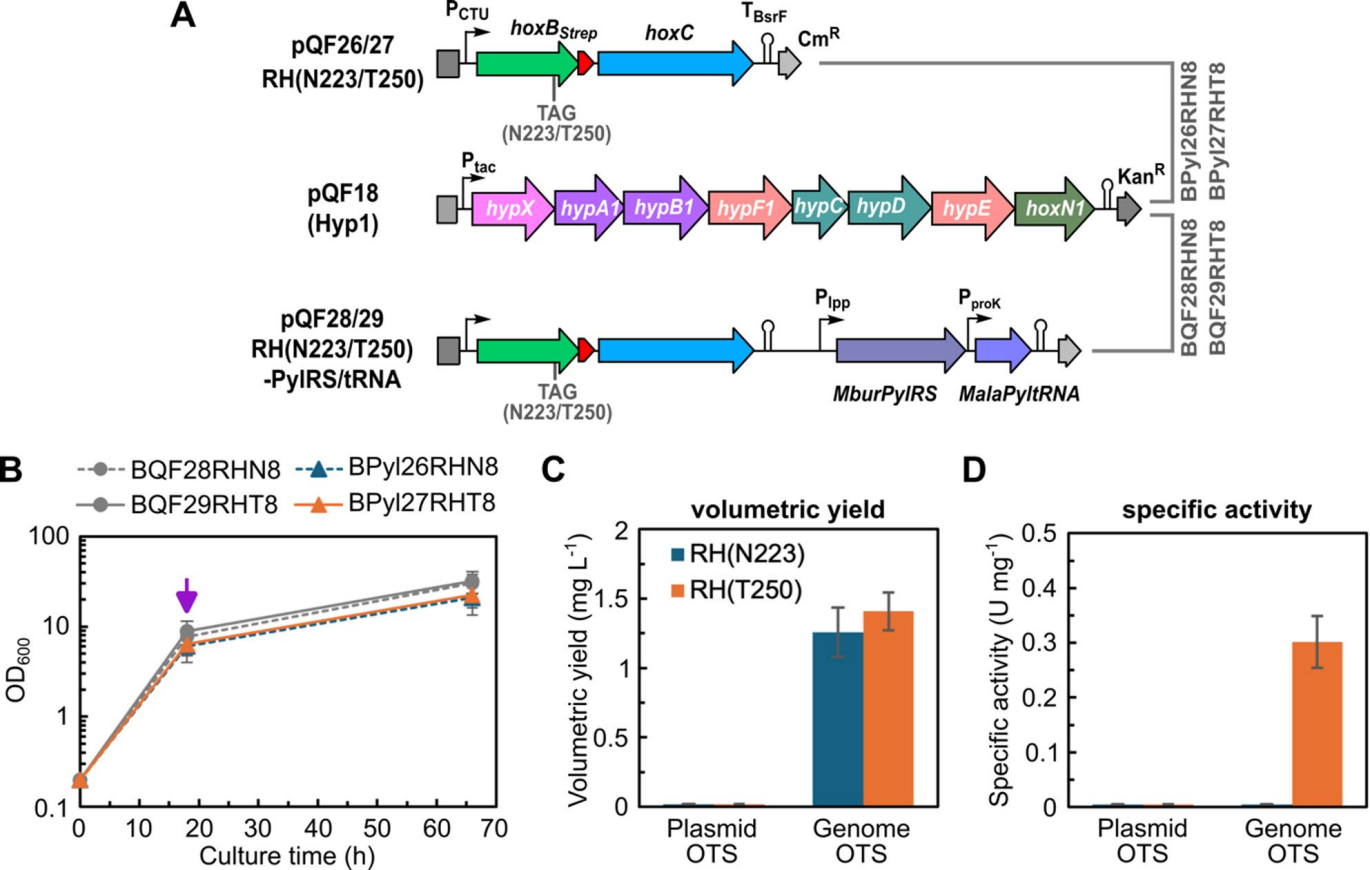
Probing ncAA incorporation into heterologously produced *C. necator* RH hydrogenase. *E. coli* BL21 Gold derivatives BPyl26RHN8/BPyl27RHT8 and BQF28RHN8/BQF29RHT8 with the chromosomally integrated and plasmid-based orthogonal PylRS/tRNA pair, respectively, were cultivated in 50 mL EnPresso B medium in 250 mL UYF at 30 °C and 250 rpm. After 18 h, RH production was induced by adding 50 µM IPTG, and 0.1 mM NiSO_4_, 0.1 mM FeCl_3_, and 2 mM Sac were added for RH maturation and ncAA insertion. Cultivation was then continued at 18 °C for a further 48 h. **A** Schematic overview of the plasmids used for RH production and Sac insertion in the different strains. **B** Growth curves for different stains. The induction time is indicated by a purple arrow. **C** Volumetric yield and **D** specific activity of Sac-labeled full-length RH variants, determined by SDS-PAGE and in vitro activity measurements

Although the CRISPR-based OTS integration into genomes is well established, the use of OTS to incorporate ncAA into complex metalloenzymes such as [NiFe]-hydrogenase remains a challenge from both a technical and a metabolic standpoint. To test the functionality of the chromosomally integrated orthogonal PylRS/tRNA system in incorporating the ncAA Sac into the small subunit HoxB of the *C. necator* RH, the plasmids pQF26 (encoding RH with N223*) and pQF27 (RH with T250*) were transformed together with pQF18 (harboring the RH maturation genes) into *E. coli* BPylRS1, resulting in the two RH production strains BPyl26RHN8 (pQF26 and pQF18) and BPyl27RHT8 (pQF27 and pQF18). First, we compared the growth of these strains in EnPresso B medium with that of the corresponding strains carrying the PylRS/tRNA pair plasmid-based (BQF28RHN8 and BQF29RHT8) instead of genome-integrated (Fig. 4A). All strains showed similar growth even after IPTG induction (Fig. 4B), indicating that the genomic integration of the PylRS/tRNA pair did not have any pronounced negative effects. Remarkably, RH, consisting of the subunits HoxC and HoxB (with either N223* or T250*) could only be purified from the *E. coli* BPylRS1 background by Strep-Tactin affinity chromatography, whereas neither HoxC nor HoxB was detected after purification from the strain harboring the plasmid-based OTS (Figure S10A, B). Compared to common aminoacyl-tRNA synthetases, PylRS has a lower catalytic efficiency, resulting in lower amounts of full-length target protein due to premature translation termination [23, 79–81]. Although engineered OTSs are often efficient enough to suppress in-frame stop codons [82–84], we observed the premature termination in both genome-integrated and plasmid-based OTSs (Figure S11A). However, with the plasmid-based OTS, exclusively truncated HoxB protein was obtained (Figure S11B), which explains why its purification failed, as the Strep-tag II is located at the C terminus of HoxB. It is tempting to speculate why stop-codon suppression is only possible with the genome-integrated OTS. *E. coli* B strains contain the release factor 1 (RF1), but its activity can vary depending on the context. During translation, RF1 competes with the orthogonal tRNA for binding to UAG, subsequently activating hydrolysis of peptidyl-tRNA to release the polypeptide and terminate translation, which impairs ncAA incorporation efficiency [85–89]. In the absence of RF1, aminoacylated orthogonal tRNAs can more effectively suppress UAG codons, thereby enhancing ncAA incorporation and increasing the yield of full-length protein [56, 90, 91]. Thus, when the catalytic activity of the OTS is too low to supply the cell with sufficient amounts of ncAA-charged orthogonal tRNA, the binding capacity of the Sac-charged tRNA is greatly compromised, resulting in predominantly truncated target protein [25, 92–94]. The superior performance of the genome-integrated OTS suggests that, despite its lower copy number, chromosomal integration provides a more stable and balanced expression level of the PylRS/tRNA components. This stability likely optimizes the ratio of aminoacylated tRNA to RF1, thereby facilitating the successful synthesis of the complex metalloenzyme RH, which is obviously not achieved by the plasmid-based system.

We obtained comparable amounts of the two full-length RH variants from *E. coli* BPylRS1 (Fig. 4C). However, immunological analysis with antibodies against the RH subunits revealed also substantial amounts of truncated HoxB* lacking the C-terminal Strep-tag II (~ 60 mg L^− 1^ for plasmid-based OTS and ~ 55 mg L^− 1^ for genome-based OTS) in the flow through fractions from both strain backgrounds (Figure S11). This observation is likely due to the presence of endogenous RF1 competition in both strains (see above). Thus, eliminating RF1 competition at UAG codons by using RF1-knockout strains [90, 91, 95] could be a strategy to improve production of full-length target proteins.

To investigate whether the presence of the ncAA affects the catalytic activity of the RH variants, the H_2_-mediated reduction of methylene blue by purified proteins was monitored spectrophotometrically (Fig. 4D, S10C, D). Only the RH variant in which T250 of HoxB was exchanged by Sac RH(T250Sac) exhibited detectable H_2_ oxidation activity. Interestingly, although T250 is located slightly closer to the distal [4Fe-4 S] cluster (3.9 Å) than N223 (4.5 Å) (Fig. 1A), it was the RH(T250Sac) variant that retained catalytic activity. Sequence conservation analysis of 100 HoxB homologs using WebLogo 3 (Figure S12) revealed that both N223 and T250 are highly conserved (100%) across all aligned sequences. This evolutionary invariance suggests that both residues are important for structural or functional reasons. However, the complete loss of activity that occurred when asparagine was substituted with Sac, suggests despite its greater distance from the cluster, that it plays a crucial structural role, likely by stabilizing the protein fold or the hydrogen-bonding network surrounding the distal cluster. This assumption is consistent with previous reports showing that alterations near Fe-S clusters in hydrogenases can significantly impair catalytic efficiency or completely abolish activity [96–99]. We conclude from these observations that threonine 250 is more tolerant of modification, whereas asparagine 223, is essential for maintaining catalytic competence. The specific activity of ~ 0.3 U mg^− 1^ of RH(T250Sac) was about half that of native RH, which had previously been purified from BQF8RH8 under the same culture conditions [11]. The catalytic activity of the RH(T250) indicates at least partially successful maturation and structural integrity of the variant in the presence of the genome-integrated OTS. The incomplete recovery of enzyme activity to the degree achieved with plasmid-based expression system in its native form is most plausibly due to the constraints imposed by the threonine-to-Sac substitution on optimal enzyme performance.

The successful production of this modified, active RH variant using the *E. coli* BPylRS1 strain addresses a critical limitation in the field of metalloenzyme engineering. Unlike previous reports of ncAA incorporation into relatively simple reporter proteins, the [NiFe]-hydrogenase is a complex, multi-subunit metalloenzyme, whose biosynthesis requires sophisticated maturation machinery. Our results demonstrate that the genomic integration of the OTS provides the genetic stability required for the successful integration of ncAAs into [NiFe] hydrogenase. Thus, the genetic code expansion is now also possible for highly complex metalloenzymes.

## Conclusions

In this study, the psychrophilic PylRS/tRNA system was successfully integrated into the genome of *E*. *coli* BL21 Gold, a robust and widely used platform for high-level heterologous protein production, using CRISPR/Cas9-mediated gene editing. The resulting recombinant strain, *E. coli* BPylRS1, enabled amber suppression-dependent production of RH variants incorporating the clickable ncAA Sac. In contrast to the plasmid-based OTS, which predominantly yielded truncated products due to premature translation termination, the genome-integrated OTS supported efficient UAG suppression and successful purification of the full-length metalloenzyme. This disparity provides direct evidence that genomic integration overcomes the traditional bottlenecks associated with plasmid-based translation systems. By positioning a Strep II-tag downstream of the amber codon, we purified catalytically active RH, and circumvented the need for RF1 deletion in the robust BL21 host. At least for the production of hydrogenases, it has been shown that chromosomal integration of OTS offers a clear advantage over plasmid-based OTS production. However, the yield of Sac-carrying RH (~ 1.5 mg L^− 1^) is low compared to that of unlabeled native RH (40 mg L^− 1^) [11]. A higher number of OTS resulting from multiple genomic integration using, e.g., CAST [100] could facilitate ncAA incorporation and thus lead to higher labeled RH yields. Although T250 is located closer to the distal [4Fe-4 S] cluster than N223, it is more tolerant to modification, as only the RH(T250Sac) could be purified in an active form.

Further enhancements, including targeted RF1 deletion and chromosomal integration of the complete maturation gene cluster, are expected to transform this platform into a robust, effective system for the bio-orthogonal engineering of sophisticated metalloenzymes with tailored functionalities.

## Supplementary Information

Below is the link to the electronic supplementary material.

Supplementary Material 1

## Data Availability

All data generated or analyzed during this study are included in this published article and its supplementary information files.
