# Supplementary material for: Genomically integrated orthogonal translation system in Escherichia coli enables production of functional modified [NiFe]-hydrogenases
Source: Microb Cell Fact. 2026 Jul 6;25:152. doi: 10.1186/s12934-026-03057-z (PMC13340272; doi:10.1186/s12934-026-03057-z)
Supplement: Supplementary file 1 — Supplementary Material 1 [file 12934_2026_3057_MOESM1_ESM.docx]

**Genomically Integrated Orthogonal Translation System in *Escherichia coli* Enables Production of Functional modified** **[NiFe]-hydrogenases**

Qin Fan*, Stevanie Stevanie, Stefan Frielingsdorf, Peter Neubauer, Oliver Lenz, Matthias Gimpel*

**Supplementary Figures**

**Figure S1. Boltz-2-predicted structure of regulatory [NiFe]-hydrogenase (RH) from *C. necator* and its catalytic reaction with its substrate H_2_**. Large subunit HoxC containing active [NiFe] catalytic center is shown in blue, whereas small subunit HoxB containing three [4Fe-4S] clusters is shown in green. [4Fe-4S] clusters are shown in yellow and orange spheres. In the catalytic center (depicted as spheres) of HoxC, nickel and iron ions are coordinated by four conserved cysteine ligands (not shown), as well as by one carbon monoxide (CO) and two cyanide (CN^-^) ligands. H_2_ is split by the [NiFe] cofactor into protons and electrons. Protons are channeled away from the active site toward the surface of the RH, whilst electrons are transferred to an electron acceptor via the [4Fe-4S] clusters.


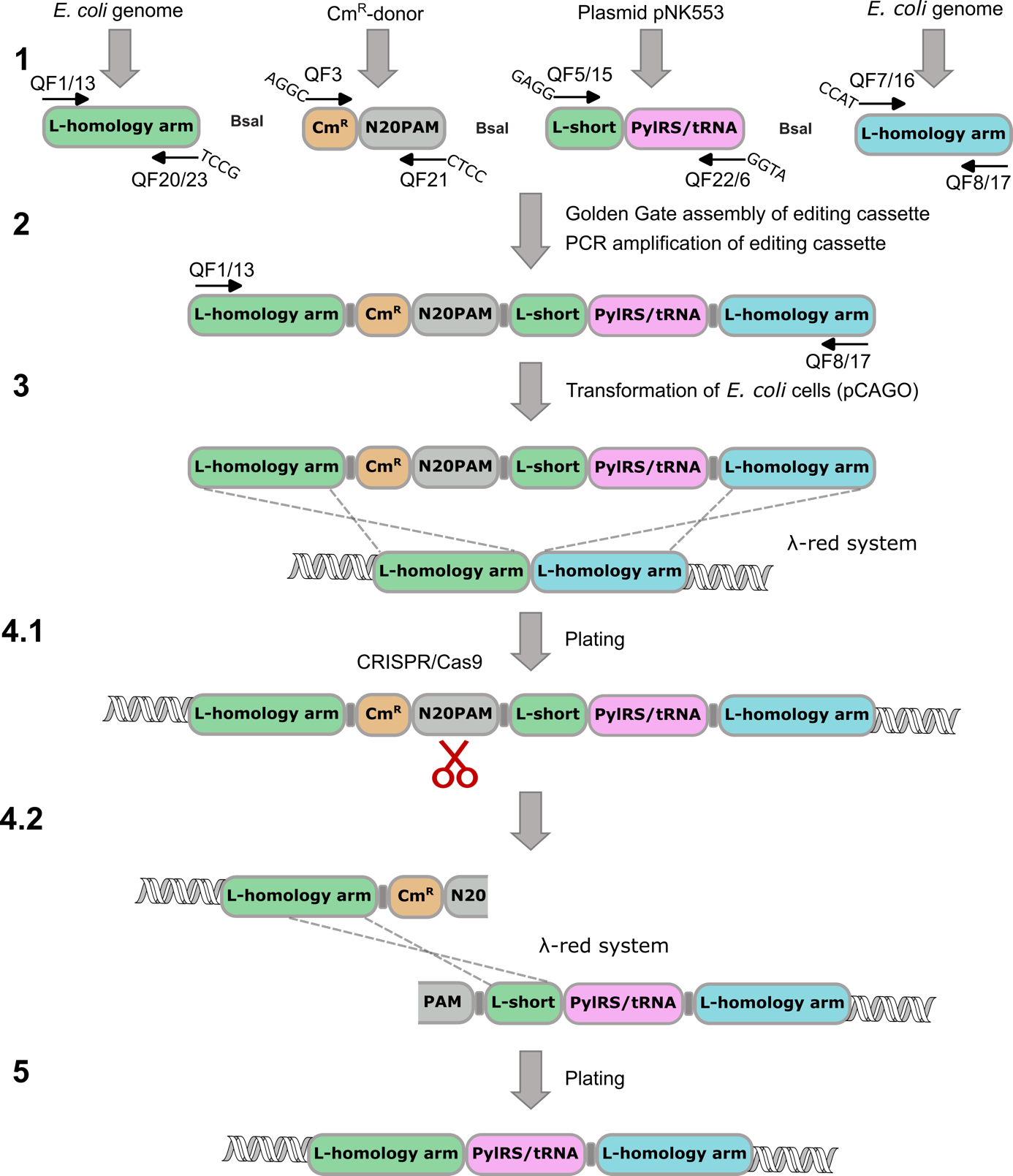


**Figure S2. Schematic overview of the CAGO-based genome integration strategy. (1)** Fragments were generated via PCR amplification and **(2)** assembled into a linear editing cassette via Golden Gate Assembly and PCR amplification. **(3)** The editing cassette is integrated into the *E. coli* chromosome via λ-red mediated homologous recombination. **(4.1)** A double strand break is induced via CRISPR/Cas9 and **(4.2)** subsequently repaired by λ-red mediated intramolecular recombination to remove the Cm^R^ from the genome, **(5)** producing the genomically modified *E. coli* strain with the desired insert at the target locus.

**
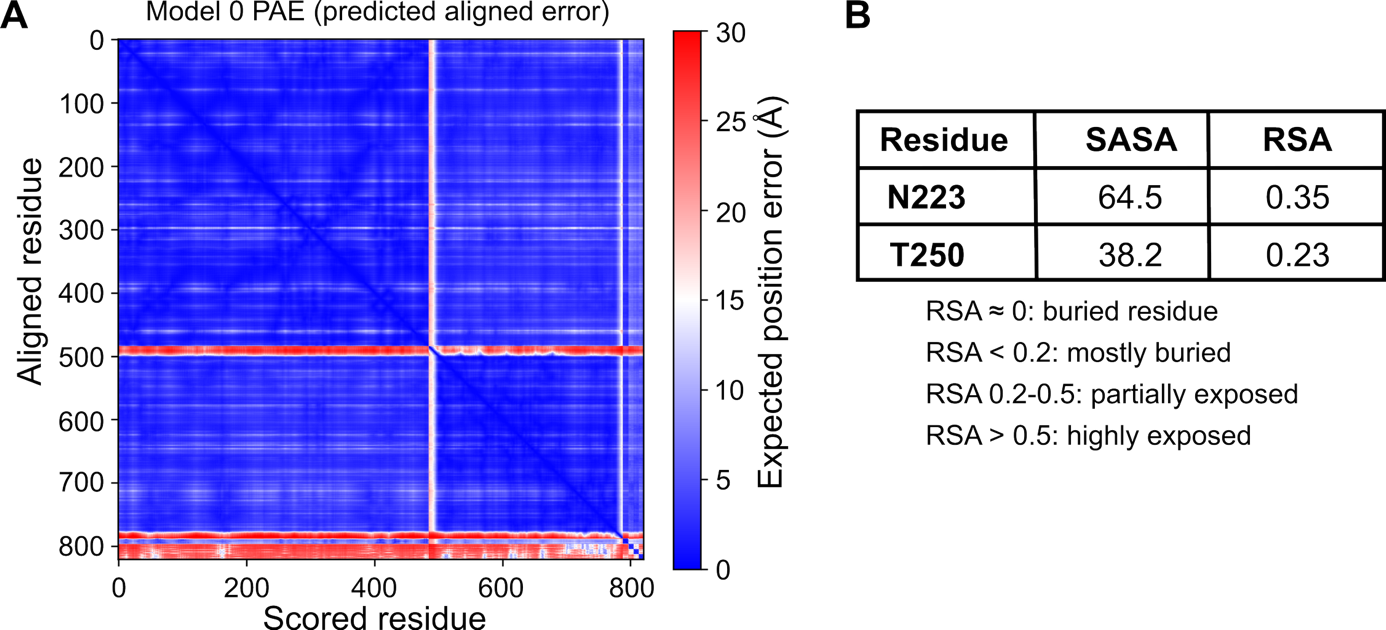
**

**Figure S3. Model quality assessment and residue accessibility analysis of RH model.** The RH model, consisting of HoxC, HoxB and its metal cofactors ([NiFe] center, three [4Fe-4S] and a single Mg^2+^ ion), was generated using Boltz-2 [1,2], which was run in a local installation at the TU Berlin math high performance cluster. (**A**) Predicted Aligned Error (PAE) plot for the Boltz-2-generated RH structural model. The color scale represents the expected error in Å, with dark blue indicating low error (high confidence). The distinct dark blue blocks along the diagonal and off-diagonal regions demonstrate high confidence in both the internal folding of the HoxC and HoxB subunits and their orientation within the heterodimer interface. This is further supported by the exceptionally high confidence scores, including a pTM of 0.96 (predicting the accuracy of the overall fold) and an ipTM of 0.95 (predicting the accuracy of the subunit interface). These metrics, combined with an overall model confidence score of 0.95, confirm the structural reliability of the model, including the positioning of the [NiFe] center, the three [4Fe-4S] clusters and the Mg^2+^ ion, which were used for subsequent distance and solvent accessibility (SASA/RSA) calculations. (**B**) Solvent accessibility parameters for targeted ncAA incorporation sites. Images and the solvent-accessible surface area as well as the relative solvent accessibility were generated or calculated using The PyMOL Molecular Graphics System, Version 3.1.8 Schrödinger, LLC. PyMol was also used to add hydrogens to residues N223 and T250 prior to calculation of SASA and RSA. Both residues exhibit RSA values > 0.2, indicating them as partially solvent-exposed confirming that these positions remain accessible for bio-orthogonal reaction with exogenous probes.


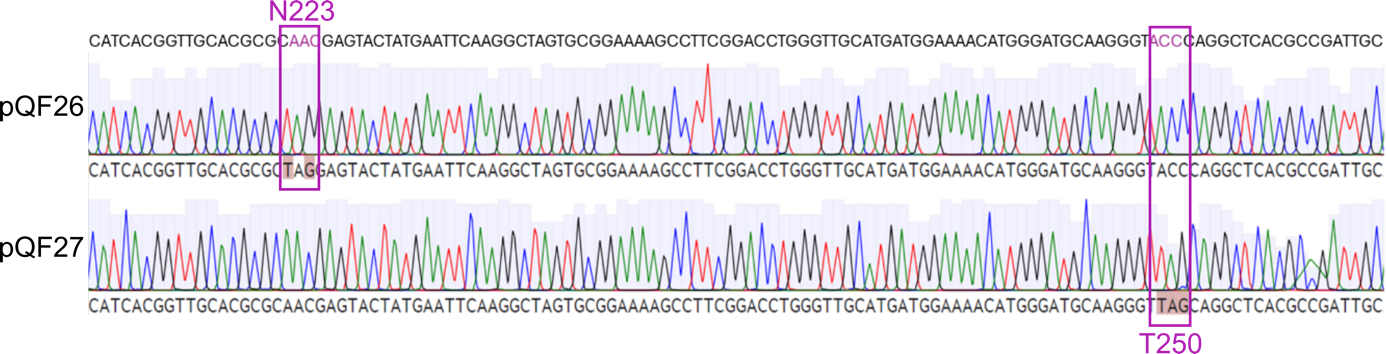
**Figure S4. Sequences of the *hoxB* regions of pQF26 and pQF27 containing the TAG mutation instead of the codons for N223 and T250.**

**
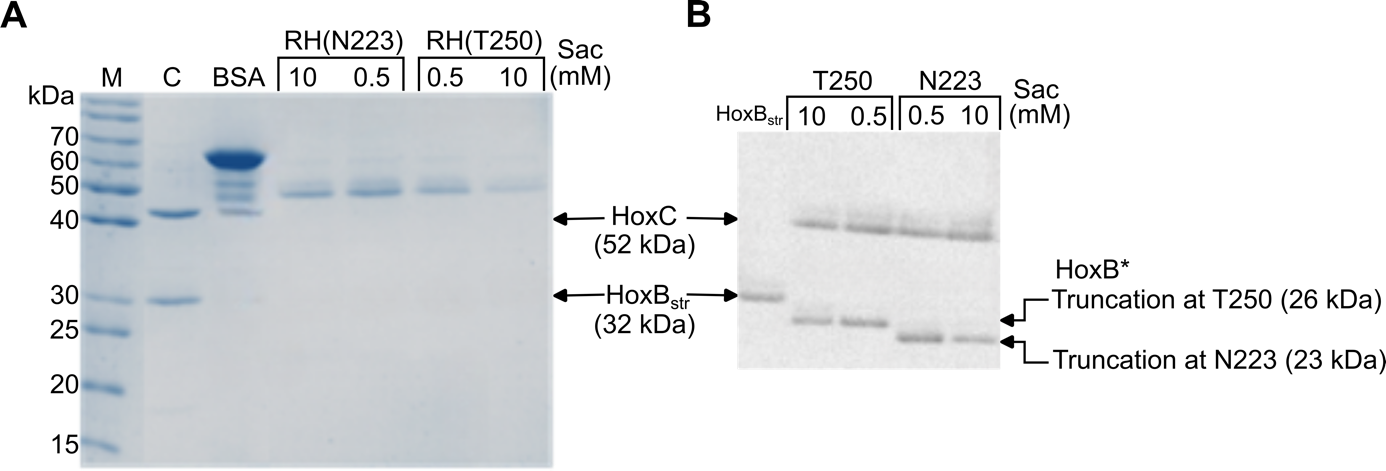
**

**Figure S5. Production of RH variants with a truncated HoxB in BL21 Gold strains.** Strains BQF28RHN8 and BQF29RHT8 were cultivated in boosted EnPresso B medium and RH production was induced by adding 0.1 mM IPTG, NiSO_4_ and FeCl_3_ together with either 0.5 mM or 10 mM Sac. After further cultivation for 48 h at 18 °C and 250 rpm, the cells were collected and the RH purified as described in Methods. (**A**) SDS-PAGE gel with the concentrated elution fractions after Strep-Tactin affinity chromatography. The RH purified from *E. coli* BQF8RH8 and BSA were used as control (C, BSA). (**B**) Western blot analysis of the Strep-Tactin column flow throughs using anti-HoxB/C antibodies (1:10000 of dilution). HoxB_strep_ purified from *E. coli* BQF4RH cultures was used as control.


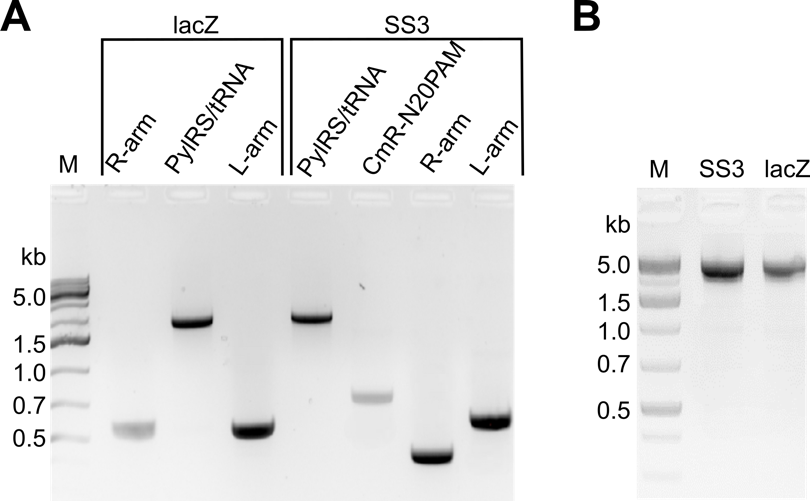


**Figure S6.** **Assembly of the linear DNA editing cassettes.** PCR-amplified fragments were used to construct linear donor DNA editing cassettes for two different genomic integration sites by Golden Gate Assembly. Four fragments were designed for each cassette. The left homology arm (L-arm), the N20PAM coupled with a chloramphenicol resistance gene (Cm^R^), L-short coupled with the PylRS/tRNA pair and the right homology arm (R-arm). While the Cm^R^-N20PAM fragment is universal for both editing cassettes the three other fragments are specific for safe site 3 (SS3) and *lacZ*, respectively. (**A**) Agarose gel (2%) showing the PCR-amplified fragments required for assembly of the two linear editing cassettes. The following fragment sizes were expected: L-arm: 553 bp and 527 bp for integration at SS3 and *lacZ*, respectively; R-arm: 413 bp and 548 bp for integration at SS3 and *lacZ*, respectively; PylRS/tRNA: 1857 bp; and CmR-N20PAM: 709 bp. (**B**) PCR amplification products after Golden Gate Assembly for integration at SS3 (3.4 kB) and *lacZ* (3.5 kB) shown in a 2% agarose gel.

**
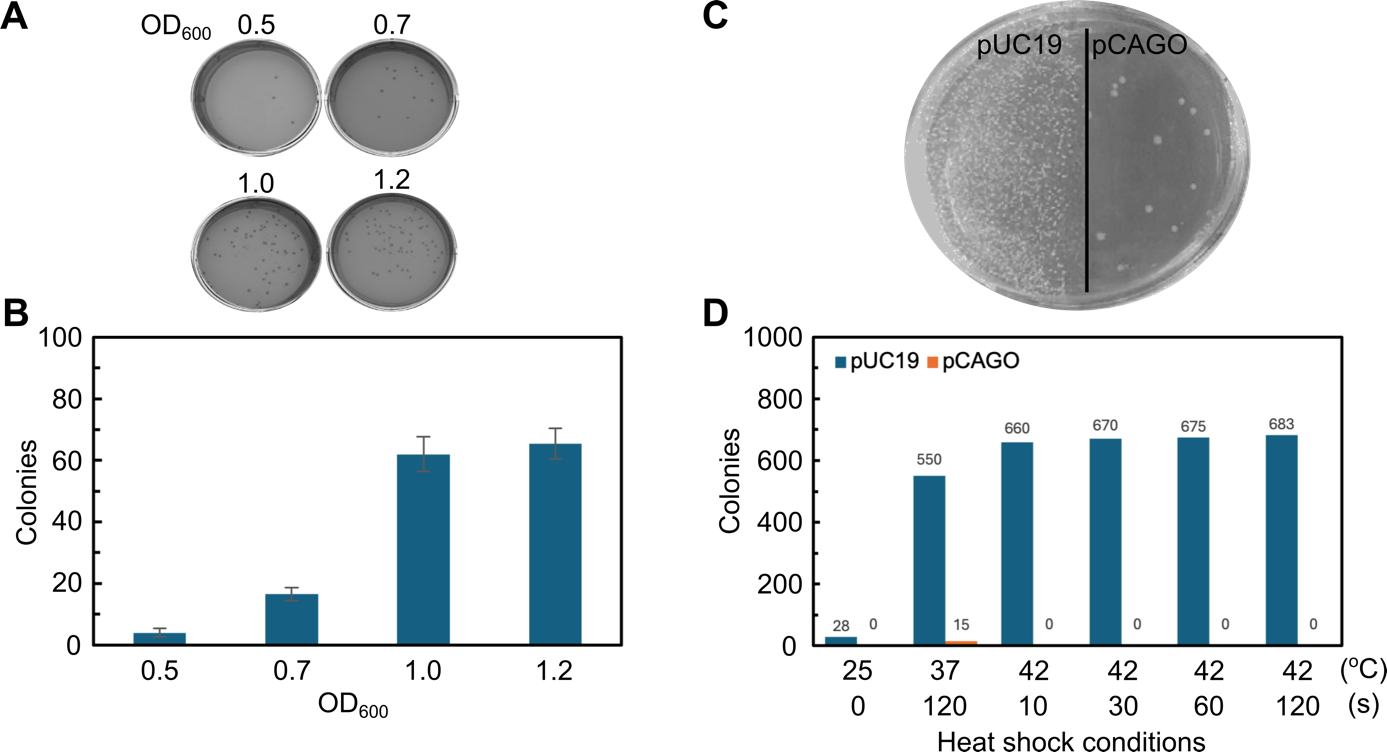
**

**Figure S7.** **Developing an efficient transformation protocol for chemically competent *E. coli* BL21 Gold cells.** *E. coli* BL21 Gold was cultivated in 10 mL TY medium at 37 °C. Cells were harvested at an OD_600_ of 0.5, 0.7, 1.0, and 1.2 and made competent using the CaCl_2_ method (see Methods). 100 µL competent cells were transformed with either 1 µL pUC19 (~100 ng µL^-1^) or 1 µl pCAGO (~200 ng µL^-1^). After heat shock at different temperatures and durations, the cells were cultivated in 1 mL TY medium at 37 °C for 1 h with gently shaking, spread onto selective TY agar plates, and incubated overnight at 37°C. **(A)** 110 µL of the transformation suspensions (10^‑1^ dilution) were plated on TY_Amp_. (**B**) Number of colonies from **(A)** grown after transformation. (**C**) Agar plate with colonies obtained from of competent cells harvested at OD 1.0, incubated with plasmids pUC19 and pCAGO and subjected to heat shock at 37°C for 2 min. (**D**) Plot of the number of colonies obtained after heat shock screening. Cells after transformation without heat shock were grown at 25 °C. In this case, the complete transformation mixture was plated.

**
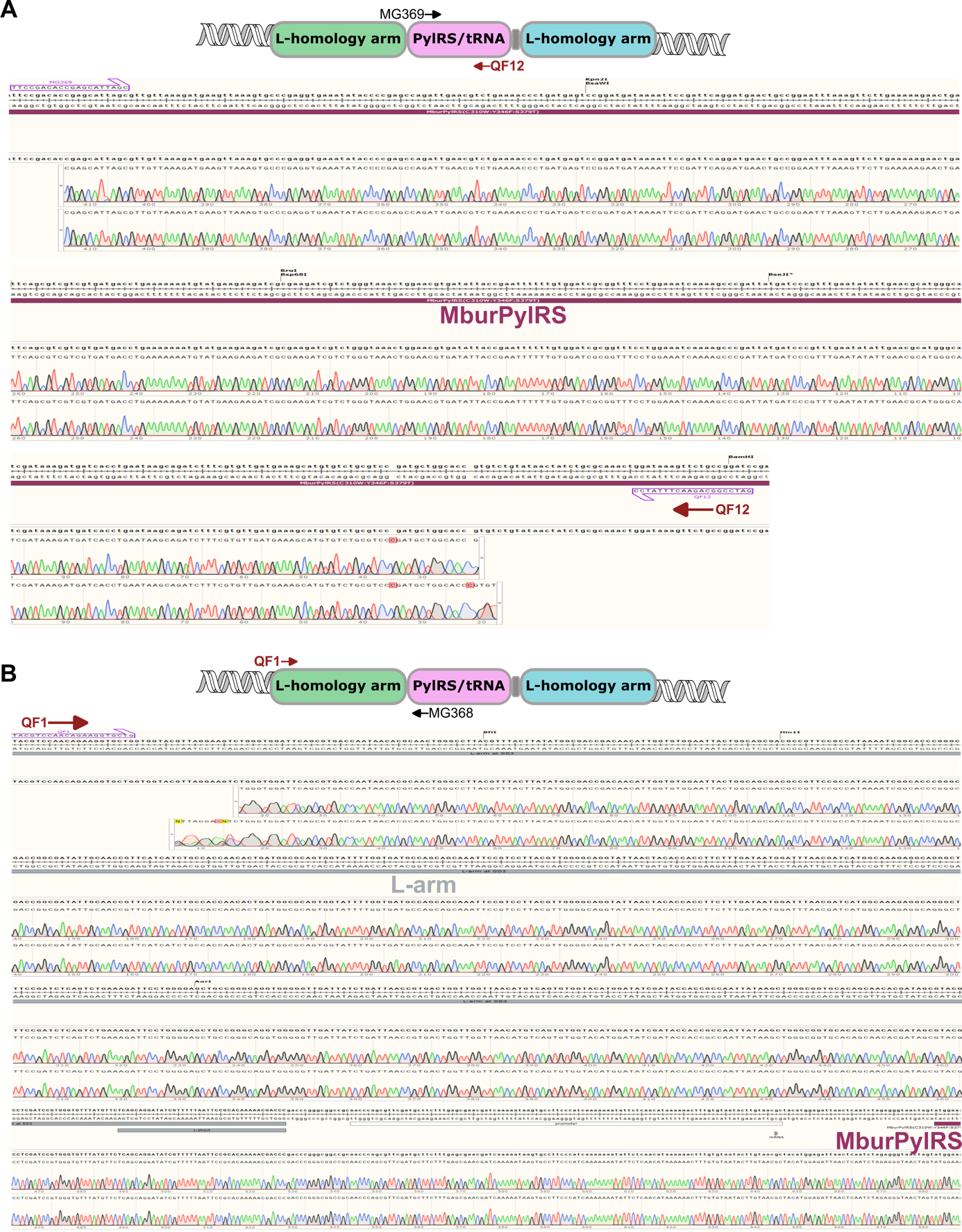
**

**Figure S8. Partial sequences of the** **DNA editing cassettes in the chromosomal SS3.** (**A**) Part of the inserted orthogonal PylRS/tRNA sequence was amplified by PCR using primers MG369 and QF12 and analyzed by Sanger sequencing using primer QF12. (**B**) Part of the inserted L-homology arm was amplified by PCR using primers QF1 and MG368 and analyzed by Sanger sequencing using primer QF1. Sequencing primers are marked in red.

**
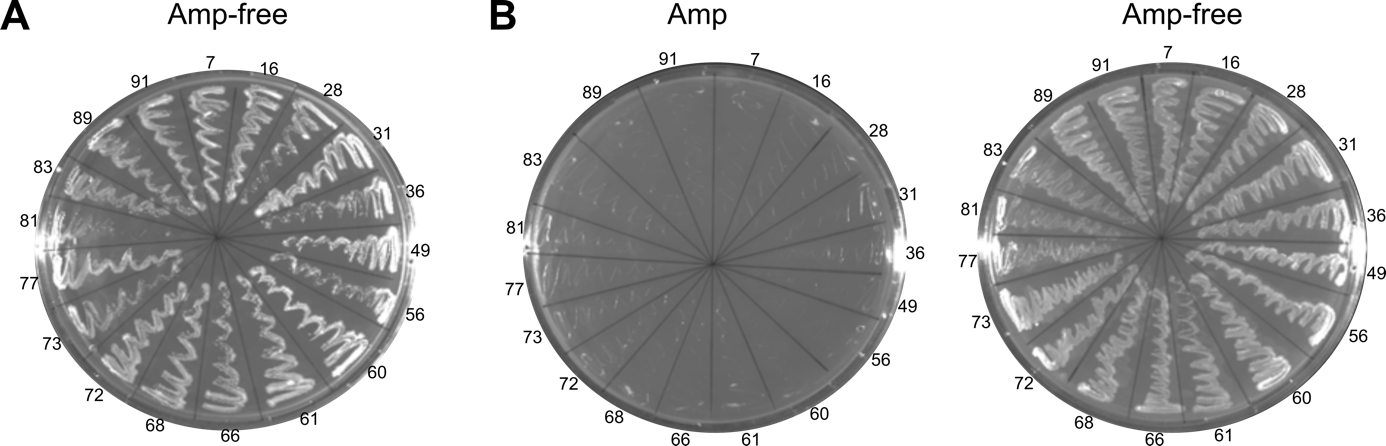
**

**Figure S9. Curing of the strains containing DNA editing cassettes from the helper plasmid pCAGO.** After genomic integration of the PylRS/tRNA pair into SS3 and subsequent removal of the Cm^R^ gene, clones were streaked on TY and TY_Amp_ agar plates and incubated at 42°C to cure the strain from the temperature-sensitive pCAGO plasmid. (**A**) After incubation for 24 h at 42°C, all clones still grew on ampicillin-free TY agar. (**B**) Clones from (A) were again plated on TY and TY_Amp_ agar and incubated for another 24 h at 37 °C. All clones were now ampicillin-sensitive indicating the loss of plasmid pCAGO.


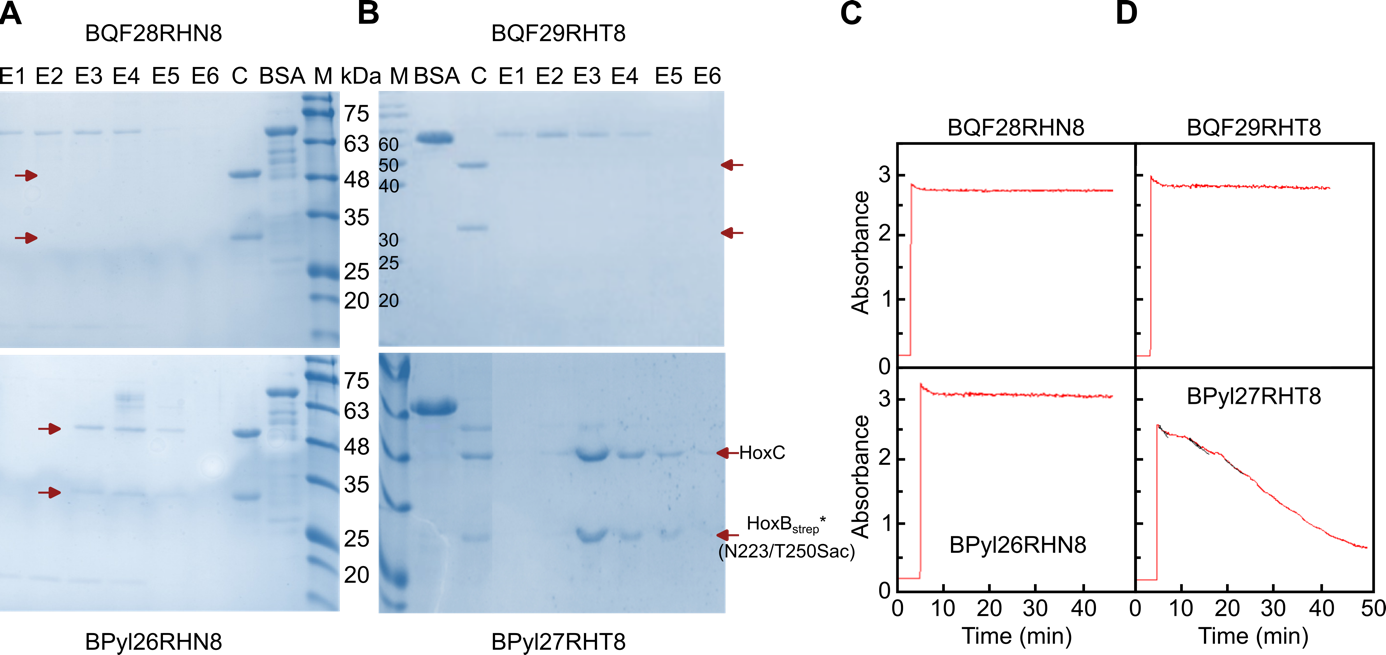


**Figure S10. Analysis of Sac incorporation into *C. necator* RH based on the genomically integrated PylRS/tRNA pair.** *E. coli* BL21 Gold strains BPyl26RHN8 and BPyl27RHT8with chromosomally integrated orthogonal PylRS/tRNA and the strains BQF28RHN8 and BQF29RHT8 with plasmid-encoded PylRS/tRNA pair were grown in 50 mL EnPresso B medium, and after 18 h of cultivation, RH production was induced by adding 50 µM IPTG, 0.1 mM NiSO_4_, 0.1 mM FeCl_3_ and 2 mM Sac. Cultivation was continued at 18 °C for another 48 h. Cells were collected by centrifugation and the RH was purified by Strep-Tactin affinity chromatography as described in Methods (**A, B**) SDS-PAGE gels showing the elution fractions of RH(N223Sac) and RH(T250Sac). BSA and native RH purified from BQF8RH8 cultures served as controls (C). The RH subunits HoxC and HoxB_strep_* are indicated by red arrows. (**C, D**) H_2_-mediated reduction activity of methylene blue by RH(N223Sac) and RH(T250Sac).


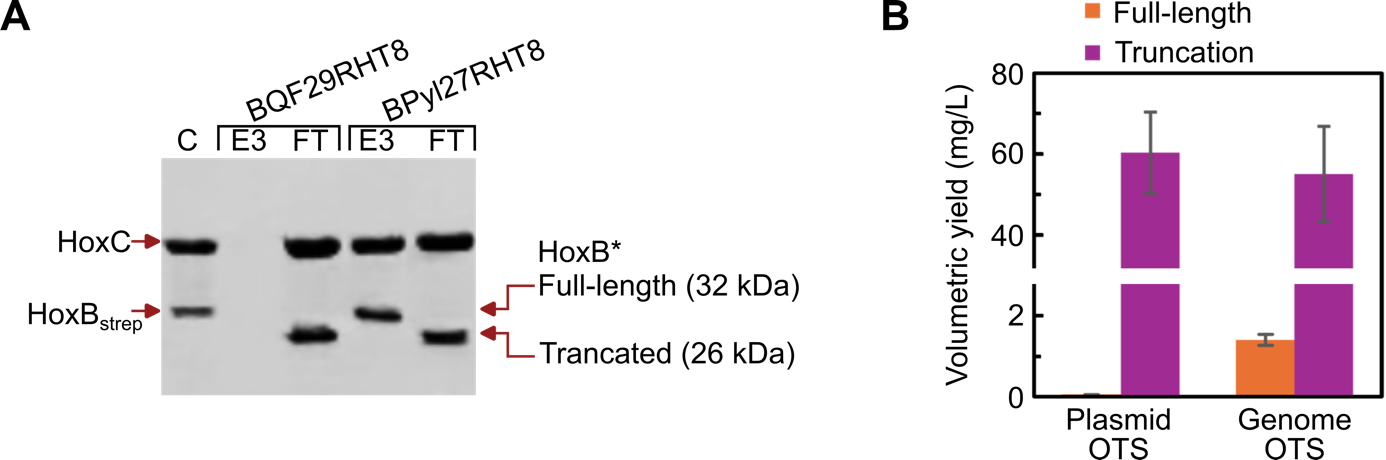


**Figure S11. Immunological analysis of RH variants produced by *E. coli* BPyl27RHT8 and BQF29RHT8.** (**A**) Western blot analysis of elution fraction E3 (Figure S9) and the flow throughs of the Strep-Tactin columns using anti-HoxB/C antibodies (1:10000 of dilution). Native RH purified from *E. coli* BQF8RH8 served as control (C). HoxB(T250Sac) (32 kDa) could be purified. Truncated HoxB (26 kDa) was found in the flow through (FT) of both soluble extracts, which could not be purified because of the missing C-terminal Strep-tag of HoxB. (**B**) Volumetric yield of full-length and truncated RH(T250Sac) determined by SDS-PAGE.

**
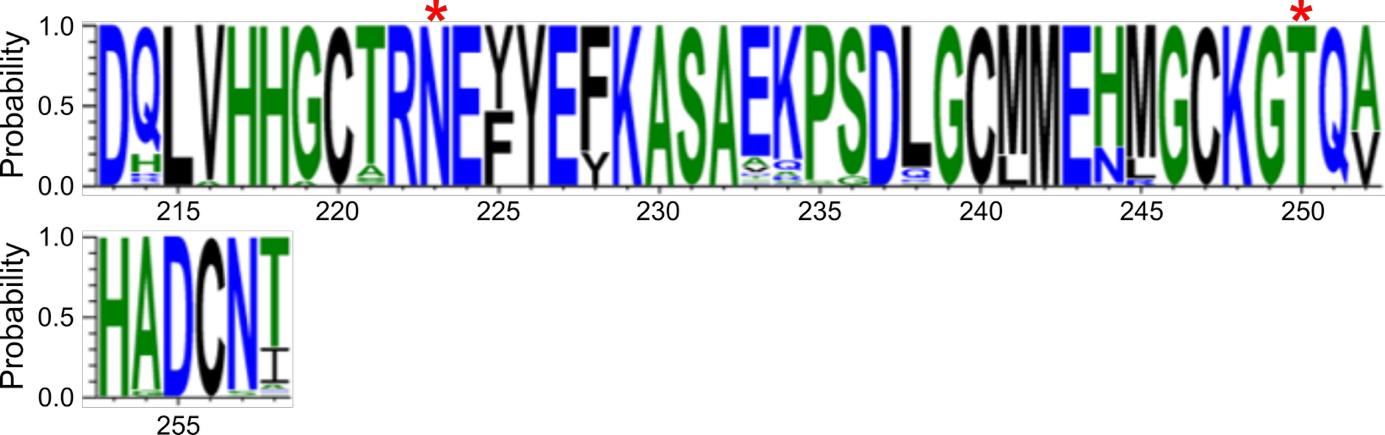
**

**Figure S12 Sequence conservation analysis of the HoxB C-terminal region.** Sequence logo plot generated using WebLogo 3[3] based on a multiple sequence alignment (MSA) of 100 HoxB sequences from diverse hydrogenase homologs. The x-axis corresponds to the residue numbering of the *C. necator* HoxB subunit. The total height of each stack represents the sequence conservation at that position (measured in bits), while the height of symbols within the stack indicates the relative frequency of each amino acid. Both targeted ncAA incorporation sites, N223 and T250, are 100% conserved across all analyzed sequences, as shown by the maximum bit score and the exclusive presence of Asparagine (N) and Threonine (T) at these positions, respectively. This evolutionary invariance may indicate a critical structural and functional role of these residues in the proximity of the distal [4Fe-4S] cluster, however, the whole region that was analyzed, revealed very low sequence diversity.

**Table S1. *E. coli* strains used in this study.**

| **Strain** | **Genotype** | **Source** |
| --- | --- | --- |
| **TG1** | *E. coli K-12, glnV44 thi-1 Δ(lac-proAB) Δ(mcrB-hsdSM)5(rK–mK–) F′ [traD36 proAB+ lacIq lacZΔM15]* | [4] |
| **BL21 Gold** | *E. coli* B, F^-^*ompT hsdS_B_*(*rB*^-^*mB*^-^) *dcm*^+^ *Tet*^R^ *gal* *endA* | Agilent, Waldbronn |
| **BL21 Gold (pCAGO)** | as BL21 Gold carrying plasmid pCAGO, Amp^R^ | This study |
| **BPylRS1** | as *E. coli* BL21 Gold with *SS3::PyrLys tRNA Synth/tRNA* | This study |
| **BPyl26RHN8** | as BPylRS1 with plasmids pQF26 and pQF18 | This study |
| **BQF27RHT8** | as BPylRS1 with plasmids pQF27 and pQF18 | This study |
| **BQF28RHN8** | as BL21 Gold with pQF28 and pQF18 | This study |
| **BQF29RHT8** | as BL21 Gold with pQF29 and pQF18 | This study |

**Table S2. Plasmids used in this study.**

| **Plasmid** | **Description** | **Reference** |
| --- | --- | --- |
| **pCAGO** | Low copy number plasmid, ori R101, temperature sensitive repA101ts, P_BAD_ - *cas9*, N20-gRNA scaffold, P_trc_ - λ‑red (Gam, Beta, Exo), Amp^R^ | [5] |
| **pNK552** | ori p15A, P_lpp_ *- Mbur*PylRS(C310W:Y346F:S379T) and P_proK_ *- Mala*tRNA_CUA_, Cm^R^ | [6] |
| **pUC19** | *E. coli* cloning vector, Amp^R^, P_lac_, MCS | [7] |
| **pQF3** | *E. coli* cloning vector, P_lac_, MCS, deletion of unique BstBI site, integration of *bsrF* transcription terminator; Cm^R^ | [8] |
| **pQF8** | as pQF3 with integration of *hoxBC* | [8] |
| **pQF18** | *E. coli, S. thermophilus* shuttle vector, P_tac_ ‑ *hypA1B1F1CDEX*-*hoxN*; Kan^R^ | [9] |
| **pQF26** | as pQF8 with mutation of *hoxB*(N223*) | This study |
| **pQF27** | as pQF8 with mutation of *hoxB*(T250*) | This study |
| **pQF28** | as pQF26 with integration of *Mbur*PylRS/tRNA | This study |
| **pQF29** | as pQF27 with integration of *Mbur*PylRS/tRNA | This study |

**Table S3. Primers used in this study.**

| **Name** | **Sequence** | **Function** |
| --- | --- | --- |
| MG358 | 5’ CAT CAC GGT TGC ACG CGC TAG GAG TAC TAT GAA TTC AAG | pQF26, HoxB(N223TAG) |
| MG47a | 5’ TTG CGG CAA TCG CCA GCC TGT | pQF26, HoxB(N223TAG) |
| MG38 | 5’ AGT TCT AGA TGG CTT GGA GGA GAA ATG AAC GCG CCT GTA TGT | pQF26, HoxB(N223TAG) |
| MG359 | 5’ CTT GAA TTC ATA GTA CTC CTA GCG CGT GCA ACC GTG ATG | pQF26, HoxB(N223TAG) |
| MG360 | 5’ AAC ATG GGA TGC AAG GGT TAG CAG GCT CAC GCC GAT TGC | pQF27, HoxB(T250TAG) |
| MG361 | 5’ GCA ATC GGC GTG AGC CTG CTA ACC CTT GCA TCC CAT GTT | pQF27, HoxB(T250TAG) |
| MG364 | 5’ TAT GAG CTC CGC AAC CCA GCG TTC GAT GCT TCT | pQF28&pQF29 |
| MG365 | 5’ AGC GAG CTC GCC ATG CAA AAA AGC CTG CTC GTT | pQF28&pQF29 |
| QF1 | 5’ GTA CGT CCA ACA GAA GGT GCT G | SS3, L-homology arm |
| QF20 | 5’ CCA GGT CTC AGC CTG GGT CGT TTT TGT GCG GAA TTA AA | SS3, L-homology arm |
| QF5 | 5’ ATA GGT CTC **TGA GGG** TCA GCA GGA TAT CGT TTT TAA TTC CGC ACA AAA ACG ACC CCG ACC CGG GCG GCG GCC GCA ACC CA | SS3, PylRS/tRNA |
| QF22 | 5’ TAG GGT CTC **GAT GG**A TGC AAA AAA GCC TGC TCG TTG | SS3, PylRS/tRNA |
| QF7 | 5’ ACC GGT CTC **GCC AT**G TAT TAT TTC TTC GCT TCT ACG | SS3, R-homology arm |
| QF8 | 5’ CTA CGC GCT CGT ATT GAA CAA C | SS3, R-homology arm |
| QF3 | 5’ GGC GGT CTC AAG GCA TGG AGA AAA AAA TCA CTG GA | SS3&Lac, CmR-N20PAM |
| QF21 | 5’ TAT GGT CTC **TCC TCG TAC TTC GGT TCG ATG**  **GAC TA**T TAC GCC CCG CCC TGC C | SS3&Lac, CmR-N20PAM |
| QF13 | 5’ TGA TGT GTC CGG CTT CTG AC | Lac, L-homology arm |
| QF23 | 5’ TAT GGT CTC **AGC CT**C CAC GCG ATG GGT AAC AGT CTT GG | Lac, L-homology arm |
| QF15 | 5’ ATA GGT CTC **TGA GGG** GTA TTT AGC GAA ACC GCC AAG ACT GTT ACC CAT CGC GTG GGA CCC GGG CGG CCG CAA CCC A | Lac, PylRS/tRNA |
| QF6 | 5’ TAG GGT CTC **GCC AT**A TGC AAA AAA GCC TGC TCG TTG | Lac, PylRS/tRNA |
| QF16 | 5’ ACC GGT CTC **GCC AT**T CTC CAG GTA GCG AAA GCC ATT TT | Lac, R-homology arm |
| QF17 | 5’ AAG CCG TTG CTG ATT CGA GG | Lac, R-homology arm |
| QF9 | 5’ ATA ACG ACG GAG CGG ATA TG | Seq. & colony PCR |
| QF10 | 5’ AGC TGA ACG GTC TGG TTA TAG G | Seq. & colony PCR |
| QF11 | 5’ AAA GCA AGC CGA ATC TAT GC | Seq. & colony PCR |
| QF12 | 5’ GAT CCG GCA GAA CTT TAT CC | Seq. & colony PCR |
| QF24 | 5’ ACA GCT CGT AGA AGG TAT ACA C | Seq. & colony PCR |
| MG366 | 5’ GCT GGC GAT TCA GGT TCA TC | Seq. & colony PCR |
| MG367 | 5’ GCA AAC AAA CCA CCG CTG GTA G | Seq. & colony PCR |
| MG368 | 5’ CTG CTA CGG CTA TTA CGA ACG G | Seq. & colony PCR |
| MG369 | 5’ TTC CGA CAC CGA GCA TTA GC | Seq. & colony PCR |

**Table S4 N20PAM similar area sequences from *E. coli* BL21 (NZ_CP053601.1) genome predicted by Cas-OFFinder.** N20PAM: 5’-TAGTCCATCGAACCGAAGTA; reverse: 5’‑TACTTCGGTTCGATGGACTA. (PCR amplification of N20PAM similar areas and sequence to check Off-target after pCAGO expression and editing experiments).

| **Number** | **Similar DNA sequences** | **Positions** | **Mismatches** |
| --- | --- | --- | --- |
| 1 | **gAGTCtATCtAtCtGAAcTATGG** CACCGGTACGCT | 916992 | 6 |
| 2 | **TAtTaCAcCGAtCCGAAGaATGG** CGCAGCGGCAAT | 2252268 | 5 |
| 3 | **TgGTtCATCaAACCGcgGaAGGG** ATCGCCATCAAG | 2252268 | 6 |
| 4 | **TgGTtCATCaAACCGcgGaAGGG** ATCGCCATCAAG | 3407697 | 6 |
| 5 | **TtcgCCATtGAACtGAAcTAAGG** TCTGGGGTTGAT | 3080086 | 6 |
| 6 | **TgGcCaATCaAACaGAAaTATGG** TCAGAAAATCTC | 4013280 | 6 |
| 7 | **TAGTtCtgCaAAaCGAAGTgGGG** ATTATTCTTCAA | 3910381 | 6 |
| 8 | **cAGatCAgCGAACCGgAtTACGG** TGCACTGCTGGA | 4475352 | 6 |
| 9 | **cAGaCCAcCaAACCcAAGTgCGG CGGCGATTAACG** | 4501009 | 6 |

**References**

1. Passaro S, Corso G, Wohlwend J, Reveiz M, Thaler S, Somnath VR, et al. Boltz-2: Towards Accurate and Efficient Binding Affinity Prediction [Internet]. bioRxiv; 2025 [cited 2026 Mar 20]. p. 2025.06.14.659707. https://doi.org/10.1101/2025.06.14.659707

2. Mirdita M, Schütze K, Moriwaki Y, Heo L, Ovchinnikov S, Steinegger M. ColabFold: making protein folding accessible to all. Nat Methods. Nature Publishing Group; 2022;19:679–82. https://doi.org/10.1038/s41592-022-01488-1

3. Crooks GE, Hon G, Chandonia J-M, Brenner SE. WebLogo: A Sequence Logo Generator. Genome Res. 2004;14:1188–90. https://doi.org/10.1101/gr.849004

4. Baer R, Bankier AT, Biggin MD, Deininger PL, Farrell PJ, Gibson TJ, et al. DNA sequence and expression of the B95-8 Epstein - Barr virus genome. Nature. Neuroscience Research Program Press; 1984;310:207–11. https://doi.org/10.1038/310207a0

5. Zhao D, Feng X, Zhu X, Wu T, Zhang X, Bi C. CRISPR/Cas9-assisted gRNA-free one-step genome editing with no sequence limitations and improved targeting efficiency. Scientific Reports. Nature Publishing Group; 2017;7:1–9. https://doi.org/10.1038/s41598-017-16998-8

6. Koch NG. Improved Pyrrolysyl-tRNA Synthetase Derived Orthogonal Translation Systems [Dissertation]. [Berlin]: Technische Universität Berlin; 2023.

7. Norrander J, Kempe T, Messing J. Construction of improved M13 vectors using oligodeoxynucleotide-directed mutagenesis. Gene. 1983;26:101–6. https://doi.org/10.1016/0378-1119(83)90040-9

8. Fan Q, Caserta G, Lorent C, Lenz O, Neubauer P, Gimpel M. Optimization of culture conditions for oxygen-tolerant regulatory [NiFe]-hydrogenase production from Ralstonia eutropha H16 in Escherichia coli. Microorganisms. Multidisciplinary Digital Publishing Institute; 2021;9:1195. https://doi.org/10.3390/microorganisms9061195

9. Fan Q, Caserta G, Lorent C, Zebger I, Neubauer P, Lenz O, et al. High-yield production of catalytically active regulatory [NiFe]-hydrogenase from Cupriavidus necator in Escherichia coli. Frontiers in Microbiology. Frontiers Media SA; 2022;13:894375. https://doi.org/10.3389/fmicb.2022.894375
